# Ectopic right coronary artery arising from the left sinus of Valsalva: A rare variant

**DOI:** 10.1002/ccr3.4996

**Published:** 2021-10-26

**Authors:** Mohamed Badri, Nora Qassem, Awadalkareem M. A. Mohammed, Moh.Mah.Fadel Allah Eljack, Khabab Abbasher Hussien Mohamed Ahmed, Osman Kamal Osman Elmahi, Mohammed Eltahier Abdalla Omer

**Affiliations:** ^1^ Interventional Cardiologist Omdurman Heart Centre Omdurman Teaching Hospital Omdurman Sudan; ^2^ Wad Medani Center for Heart Disease and Surgery Wad Medani Sudan; ^3^ Wad Medani Center for Heart Disease and Surgery MBBS University of Bakht Alruda Wad Medani Sudan; ^4^ Faculty of Medicine University of Khartoum Khartoum Sudan; ^5^ Faculty of Medicine Ibn Sina University Khartoum Sudan; ^6^ Internal Medicine Department Faculty of Medicine and Health Sciences Gadarif University Gadarif Sudan

**Keywords:** cardiology, ectopic coronary artery

## Abstract

An ectopic coronary artery is observed in 0.05–0.1% of angiographic series, may be of tangential origin or proximal endocardial pathway, may result in a slit hole that interferes with flow, and is associated with sudden death.

## Graphical abstract

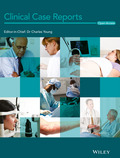

## 1 INTRODUCTION

Ectopic origin of the right coronary artery from the left sinus of Valsalva is an extremely rare anomaly of coronary arterial morphology, often incidentally discovered and clinically silent. We, hereunder, experienced a case of ectopic origin of the right coronary artery from the left sinus of Valsalva.

Congenital anomalies and variations of the coronary arteries (CAA) are present at birth but can remain symptomatically silent for life, being discovered accidentally during interventional radiological procedures or whose discovery is prompted by cardiac chest pain. In the published note, their overall prevalence is approximately 1.3%. CAAs can be benign or malignant. Benign variations include 1) origination of the left anterior descending and left circumflex arteries from the left sinus of Valsalva (LSV), 2) the circumflex artery originating from the right coronary artery or right sinus of Valsalva (RSV), and 3) ectopic origin of the right coronary artery from the ascending aorta.^1^ The most common malignant variation of the coronary arteries is the ectopic origin of the right coronary artery from the LSV, which demonstrates a single coronary artery, beginning from the aortic trunk via a single coronary arterial ostium, acting as the primary blood supply to the heart.^2^ Here, we present a case of ectopic origin of the right coronary artery from the left sinus of Valsalva.

## 2 CASE REPORT

A 35‐year‐old male laborer was admitted following a one‐month history of chest pain, including two weeks of dizziness and recurrent syncopal attacks. History taking demonstrated an extensive past medical history of chest pain since childhood and a positive family history of sudden death and sickle cell disease. Physical examination showed no remarkable findings, which was followed by a full blood count, serum troponin, urea, and electrolytes—all within normal range. A 24‐hour electrocardiogram (ECG) and echocardiogram were then performed, respectively portraying a normal sinus rhythm and normal cardiac structure with good ventricular function. A coronary angiogram was then performed using Judkin's catheterization technique, which revealed an ectopic origin of the right coronary artery from the LSV. Following this diagnosis, the patient was referred to surgery for a coronary artery bypass graft (CABG), in which the right coronary artery was found to originate between the aorta and the pulmonary trunk. Post‐operative complications included moderate anemia (Hb: 9.3g/dL) and cellulitis, treated with 4 units of blood and benzylpenicillin, respectively. The patient was discharged on analgesia (Paracetamol 1 g PO QDS) and antibiotics (Amoxicillin/clavulanic acid 1g BD for 7 days). Following up 7 days post‐operatively, no further episodes of chest pain or syncope were reported by the patient.

## 3 DISCUSSION

CAAs are rare cardiac anomalies, with an incidence rate of <1% and are often misdiagnosed as sickle cell crisis in patients with a history of sickle cell disease or trait.^3^ With the exception of a coronary artery traversing between the aorta and pulmonary trunk potentially causing sudden death at a young age due to extrinsic coronary occlusion, CAAs usually carry minimal clinical significance. In a study conducted by Taylor et al it was demonstrated that 25% of CAAs culminated in asymptomatic sudden death, with autopsies showing evidence of repetitive episodes of minor myocardial infarctions—diffuse necrosis and myocardial fibrosis.^4^ Obstruction of blood flow can be due to multiple causes—1) obstruction of the coronary ostium due to slit‐like morphology, 2) the aorta and pulmonary trunk compressing the right coronary artery, and 3) aortic and pulmonary arterial distension leading to stretching and increased tension of the right coronary artery.^5^ CT coronary angiogram is advantageous over ECG and conventional coronary angiography as it detects not only the extent and site of stenosis but also the ectopic origin and course of the anomalous coronary artery. An increase in CAAs has been diagnosed since the advent of CT coronary angiography.^6^

## 4 CONCLUSION

Coronary artery anomalies can remain clinically silent and are usually diagnosed incidentally or their diagnosis is driven by cardiac events. Misdiagnosis can be an easy pitfall in sickle cell disease, and thus, CAAs can be easily missed. It is believed that despite normal troponin and blood parameters, CT coronary angiogram was paramount to accurately diagnosing the case.

## CONFLICT OF INTEREST

The authors have no conflict of interest to declare.

## AUTHOR CONTRIBUTIONS

EIA, the first author, collected the data, analyzed the results, and wrote the manuscript. NQ, AMA, FAE, KH, OKE, and MEO wrote the manuscript, revised the manuscript, and did editing. All authors read and approved the final manuscript.

## ETHICS APPROVAL

Not applicable.

## CONSENT TO PARTICIPATE

Verbal consent and written consents were obtained from the patient before writing the case or using investigations.

## CONSENT

Written consent to publish this information was obtained from the patient. The patient gave written consent for his personal clinical details along with his MRI to be published in this study. This patient has not been reported in any other submission by the authors or anyone else.

## Data Availability

The datasets used and/or analyzed during the current study are available from the corresponding author on reasonable request.
